# A Tragical Paediatric Case History of Intraorbital and Intracranial Epithelioid Hemangioendothelioma

**DOI:** 10.1155/2012/396097

**Published:** 2012-10-24

**Authors:** K. Aniba, M. Laghmari, M. Lmejjati, H. Ghannane, S. Ait Benali

**Affiliations:** Department of Neurosurgery, Mohammed VI University Medical Centre, and Cadi Ayyad University, Marrakesh, Morocco

## Abstract

Epithelioid hemangioendothelioma (EHE) is a rare tumor of intermediate malignancy. We report a case of intracranial and intraorbitar EHE. A 3-year-old girl presented with a 3-month history of progressive left exophthalmia. Neuroradiologic imaging (CT scan and MRI) showed an intraorbitar process with an intense enhancement extending to temporal fossa, ethmoidal bone, nasal fossa, maxillary sinus, and cavernous sinus. The angiogram was normal. The tumor was operated through subfrontal approach but only a partial resection was performed. The histological diagnosis was epithelioid hemangioendothelioma. The patient was neurologically intact 2 months after surgery without exophtalmia. However 4 months after surgery he displayed a fall of the right eye vision with intense headache. Control CT scan showed persistence of important tumoral residue. Epithelioid hemangioendothelioma is a hemorrhagic tumor. Total removal must be possible. Otherwise, we recommend a complementary chemoradiotherapy and close followup. We propose this interesting case history of a tragical evolution of EHE in contradiction with what has already been reported.

## 1. Introduction

Epithelioid heamangioendothelioma (EHE) is a rare vascular tumor that represents a transitional histology between a well-differentiated hemangioma and anaplastic hemangiosarcoma [1]. The term epithelioid heamangioendothelioma was proposed firstly by Weiss and Enzinger in 1982 [1, 2].

EHE occurs usually in the lung, liver, and long bone. Cranial EHE is very rare. The tumor can occur at any age and does not show sexual predominance [3]. We report the illustrative case of a 3-year-old girl harbouring primary intracranial hemangioendothelioma and we describe the clinical history and radiological and pathologic features of this rare entity.

## 2. Case Report

**Figure 1:**
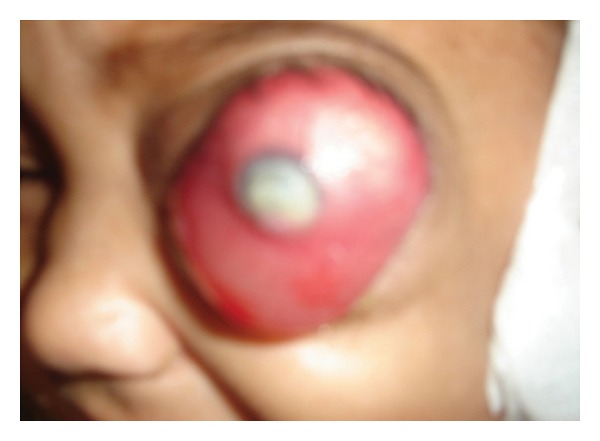
Photograph taken in the second hospitalisation shows exophtalmia.

A 3-year-old girl without pathological antecedents presents with a one-month history of exophthalmia (Figure 1) with signs of raised intracranial pressure (cephalalgia and vomiting). Physical and neurological examination revealed left exophthalmia without any vascular sign, a convexity of the palate, and left temporal optical atrophy.

**Figure 2:**
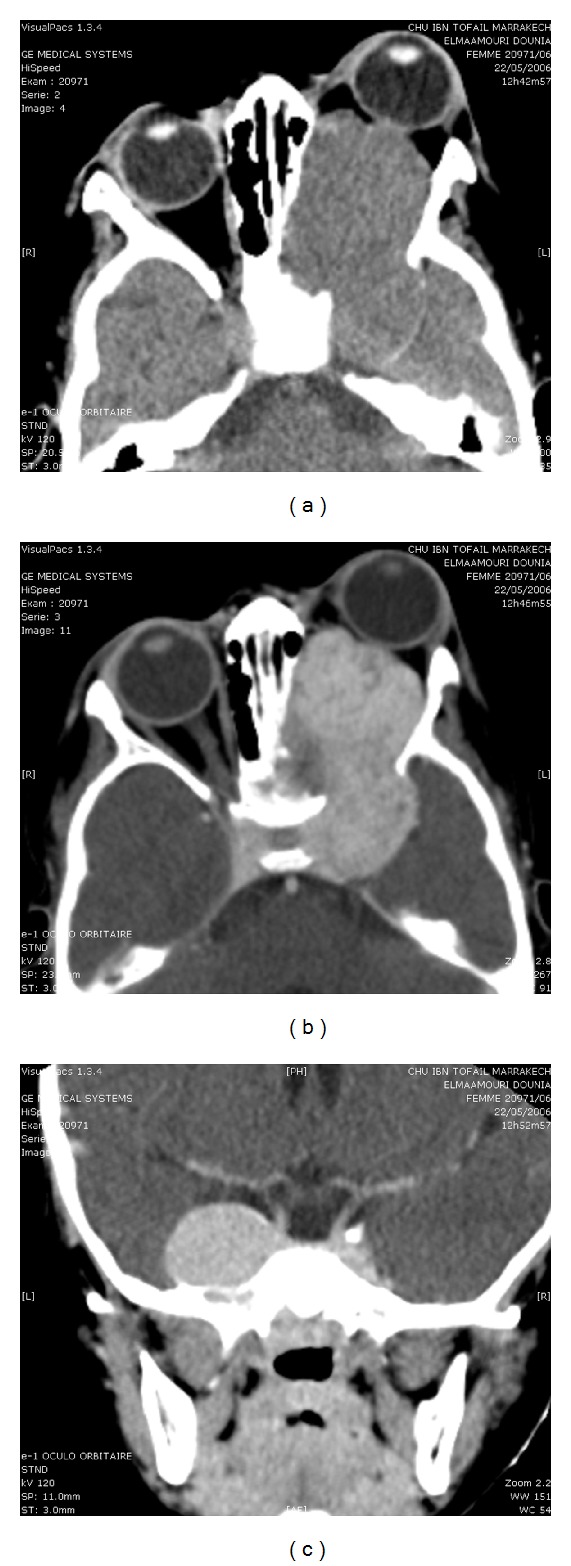
CT scan on axial (a) and coronal (b) views with contrast administration shows an endoorbital tumor involving the optical nerve spreading to the cavernous sinus and widening the pterygopalatine fissure.

**Figure 3:**
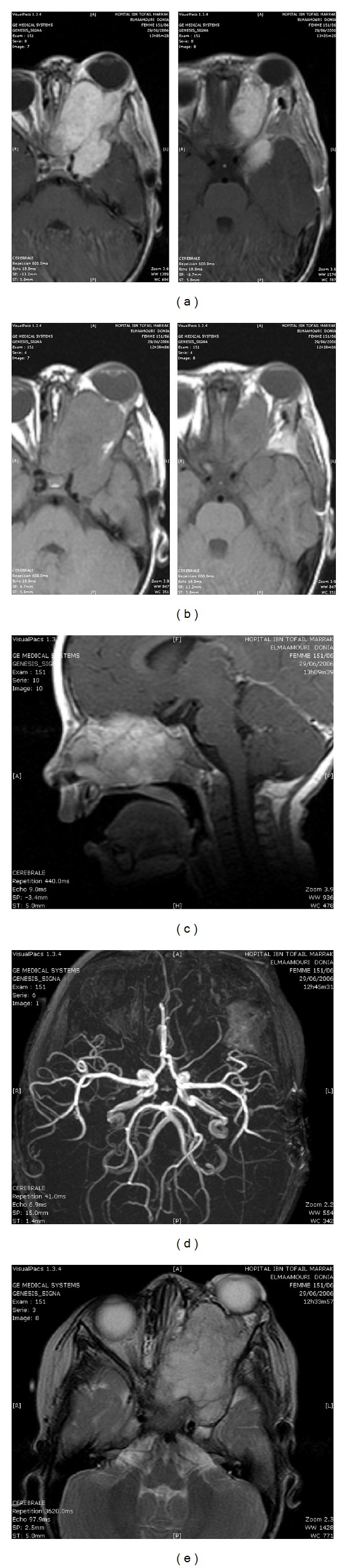
Magnetic resonance imaging in the axial (a, b, and e) and sagittal (c) planes showing the tumoral process in low signal in T1 (b) and high signal in T2 weighted images (e) as well as intense and homogeneous enhancement after contrast administration (a, c). The angio RMI sequence (d) shows the vascular character of the tumor (d).

Axial and coronal CT scan through the left orbit with contrast (Figure 2) shows an endoorbital tumor involving optic nerve, spreading to the cavernous sinus and widening the pterygopalatine fissure. Magnetic resonance imaging (MRI) (Figure 3) in the axial, sagittal, and coronal planes of the left orbit shows the tumoral process with low signal T1, high signal T2, and flair weighted sequence as well as intense and homogeneous enhancement after contrast administration. The angiogram sequences show vascular signs of the tumor.

A surgical resection with cranioorbital approach was performed; it consisted of left orbital exenterating and partial resection of the nasal and cranial extension. The peroperative finding was a reddish solid tumor. Postoperative course was uneventful.

**Figure 4:**
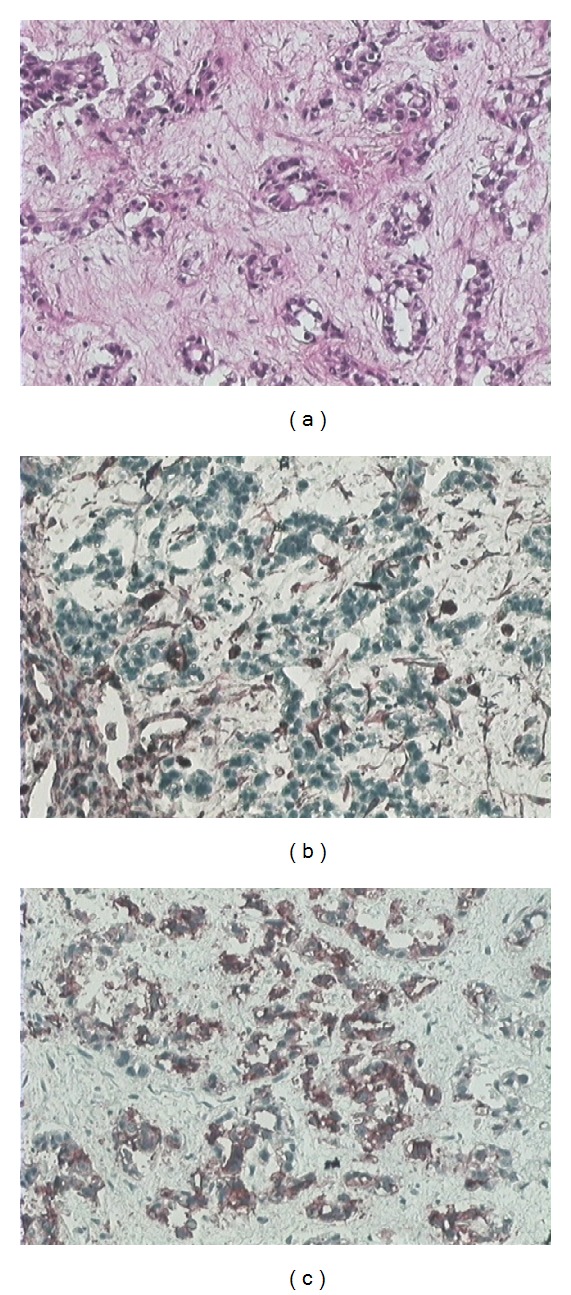
(a) Histopathologic examination revealed pathognomonic features of a hemangioendothelioma, showing that the tumour was mainly composed of epithelioid cells which form primitive vascular channels. Some cells even showed evocating intracytoplasmic vacuoles. (b, c) Immunohistochemical stains for factor CD 34 and vimentin displayed positive reaction.

Histopathologic examination (Figure 4) revealed characteristic features of an haemangioendothelioma. Microscopically, the neoplastic cells were epithelioid with growing tubules corresponding to primitive vascular channels. Cytoplasm contains large vacuoles sometimes from one to two erythrocytes. Some focal areas of necrosis could be seen. The proliferation index was elevated. The immunohistochemical analysis was positive for vimentin, CD-34.

**Figure 5:**
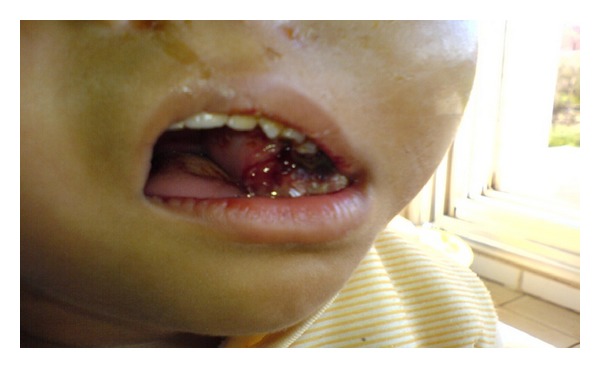
Photograph taken in the second hospitalisation shows the recurrence of the tumor.

A therapeutic complement with radiotherapy base and chemotherapy is proposed but the patient relatives refused. Two months later, the patient presented blindness of the right eye and an appearance of the tumor through the left orbit and the oral cavity (Figure 5). The patient was admitted in the reanimation service and died after four days from her hospitalization (2 months 15 days after the diagnostic).

## 3. Discussion

Epithelioid hemangioendothelioma is a rare vascular tumor of intermediate malignant features within the progression of hemangioma to angiosarcoma [4] and relatively favourable prognosis. The main occurrence sites are soft tissues, liver, lung, and bone [1].

Weiss and Enzinger described the first cases and proposed the term epithelioid hemangioendothelioma in 1982 [2], they described four variants of hemangioendothelioma including epithelioid, spindle cell, papillary, and kaposiform [2, 5]. Of these variants only the epithelioid one has been described to occur in the cranial cavity [5].

The tumor can occur at any age and does not show sexual predominance [3]. Concerning our patient, the diagnosis of EHE was formulated on the basis of the microscopic characteristics and immunohistochemical reactivity. The epithelioid aspect of the tumoral cells, the presence of intracytoplasmic vacuoles as a sign of vascular neoformation, and the atypical disposition in cords and nests in a myxoid stroma are all included in the classical description of EHE [1, 6]. Immunoreactivity to factor VIII-related antigen and ulex europaeus are specific for an epithelial tumor [3, 5, 7, 8].

The hypercellularity and the poor vascular differentiation of the tumor eliminate a diagnosis of hemangioma, whereas the low mitotic index and the absence of necrotic foci role out a diagnosis of angiosarcoma.

The exact prognosis of EHE was not established clearly. Weiss et al. have reviewed the biological behaviour of 46 cases in soft tissue, lung, liver, and bone. They reported a recurrence rate of 13%, a metastasis rate of 31%, and a mortality rate of 13%, all within a 4-year period [1, 9]. However there are reported patients with EHE that, although histologically benign, producted metastasis 10 years after the first operation [2].

Treatment of EHE depends on its location and degree of malignant potential. It consists of surgical resection. Wide local excision and possible regional lymph node resection are advocated because the lymph node is a common metastasis site [1, 10]. Radical excision and close followup are the main stones of current standard therapy. Chemotherapy and radiation therapy were not considered effective on EHE [11]. Some authors recommend that preoperative embolisation should be an essential procedure to ovoid massive intraoperative bleeding [5]. Because of the fast dramatic evolution, we recommend a close followup and chemoradiotherapy mandatory in the case of incomplete removal.
